# Differential Modulation of Copper(II) Interactions with the 18–22 Coordinating Amylin Fragment by the Geometric Isomers of a New Nicotinoyl Hydrazone: A First Study

**DOI:** 10.1021/acsomega.5c04850

**Published:** 2025-07-11

**Authors:** Alessandra Carvalho, Karina C. Pougy, Anderson S. Pinheiro, Daphne S. Cukierman, Nicolás A. Rey

**Affiliations:** † Department of Chemistry, 28099 Pontifical Catholic University of Rio de Janeiro (PUC-Rio), Rio de Janeiro 22451-900, Brazil; ‡ Department of Biochemistry, Institute of Chemistry, 28125 Federal University of Rio de Janeiro (UFRJ), Rio de Janeiro 21941-909, Brazil; § Department of General and Inorganic Chemistry, Institute of Chemistry, State University of Rio de Janeiro (UERJ), Rio de Janeiro 20550-013, Brazil

## Abstract

Type 2 diabetes *mellitus* is a multifactorial disease associated with insulin resistance and pancreatic β-cell dysfunction. Interestingly, this disease has also been associated with the aggregation of islet amyloid polypeptide (IAPP or amylin). This peptide can bind to physiological metal ions such as copper­(II), which enhances its aggregation and induces oxidative stress. For this reason, the use of moderate chelators constitutes a compelling potential therapeutic strategy. In this work, we synthesized and characterized the geometric isomers of a new compound, 1-methylimidazole-2-carboxaldehyde nicotinoyl hydrazone (**X1NIC**), aiming to evaluate their differential interactions with copper­(II) and the hIAPP_18–22_ peptide fragment. Both compounds demonstrated high aqueous stability and adequate lipophilicity for biological membrane crossing. Coordination studies revealed that both ML and ML_2_ complexes can be obtained in solution for the tridentate ligand **X1NIC-(** ***E*** **)**, with the former being more stable. On the other hand, the bidentate ligand **X1NIC-(** ***Z*** **)** only forms ML species. Both isomers effectively set up ternary complexes with Cu^2+^ and hIAPP_18–22_, altering the redox behavior of the copper-peptide system. These results, obtained through cyclic voltammetry experiments, suggest a protective effect of the ligands with respect to metal-induced oxidative stress. This study constitutes the first comparative report on the coordination and reactivity of geometric isomers of a bioactive *N*-acylhydrazone, and the findings described herein highlight this class of compounds as promising chemical tools for the modulation of abnormal metal-peptide interactions implicated in type 2 diabetes pathogenesis.

## Graphical abstract

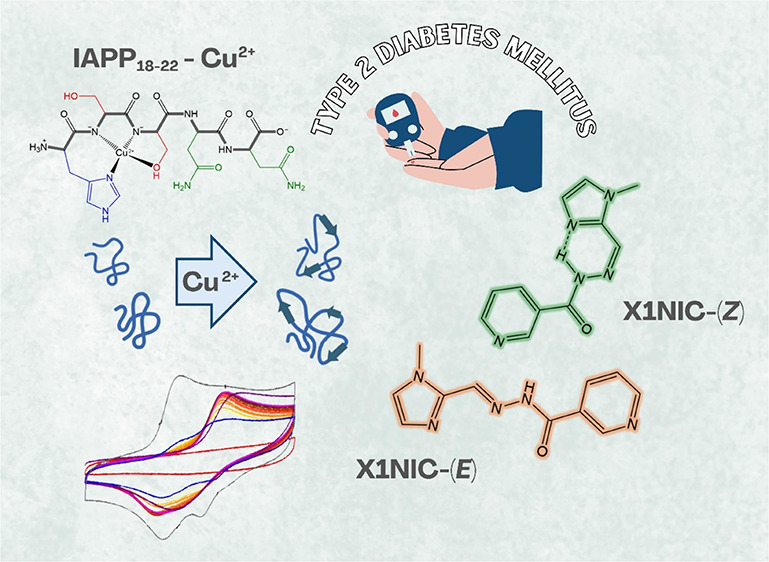

## 1 Introduction

Type 2 diabetes *mellitus* (T2DM) is a chronic endocrine disorder characterized by hyperglycemia, which may result from defects in the secretion or action of insulin.^1^ Although many patients are asymptomatic, clinical manifestations including unintended weight loss, fatigue, blurred vision, and numbness in the hands or feet are possible.

Interestingly, T2DM can also be considered an aggregopathy since it has been associated with the presence of aggregated pancreatic islet amyloid polypeptide (IAPP).^2^ Also known as amylin, physiological IAPP is responsible for both the regulation of food intake and the transmission of signals to the central nervous system.^3^ This 37-residue amino acid peptide is secreted by the β-cells of pancreatic islets^4^ and is usually present in increased concentrations in insulin resistance conditions.^5^ Amyloid deposits of IAPP constitute a histological hallmark of T2DM, found in over 90% of patients.^2,6^

As for many other aggregopathies, such as Alzheimer’s and Parkinson’s diseases, the interplay between metal ions and amyloidogenic proteins or peptides is also thought to play a significant role in the pathophysiology of T2DM, since metal binding leads to oligomerization, oxidative stress, toxicity, and cell death.^7,8^ Whether these abnormal interactions are a cause or a consequence of biometal dyshomeostasis, which is another common feature of such disorders, is still debated.^9^ In the case of T2DM, it has been shown that an imbalance of essential metals can initiate problems in the pancreatic islets, leading to the development of diabetes, and such an imbalance can lead to the production of ROS.^10^ Assays carried out in diabetic mice showed that treatment with a copper chelator decreased plasma levels of ROS, indicating its importance in the treatment of T2DM.^11^

In the context of metal-enhanced aggregopathies, we have already demonstrated that *N*-acylhydrazones efficiently inactivate aberrant metal-bound proteins and peptides, either through metal sequestering or through the formation of stable ternary complexes, and that they present good pharmacological profiles (stability, solubility, and membrane crossing) and optimized affinity that leads to low toxicity.^12–17^ Although easily obtained, *N*-acylhydrazones are not rarely observed as a mixture of geometric (*E*)- and (*Z*)-isomers, with the former being usually present at higher concentrations in solution.^13,18^ When intended as passivators in the context of metal-enhanced aggregopathies, however, the coordination particularities of each isomer must be taken into account. When the aldehyde precursor contains an aromatic ring with a heteroatom at position 2, (*E*)-isomers are typically tridentate, while (*Z*)-isomers perform only as bidentate, and potentially binucleating, chelators. Scheme 1 illustrates the coordination prospect of both geometric isomers of the new ligand 1-methylimidazole-2-carboxaldehyde nicotinoyl hydrazone (**X1NIC**), which we propose in the present study based on the promising results recently obtained by using the synthetic precursor 1-methylimidazole-2-carboxaldehyde. **X1NIC** also includes a hydrazide-derived, biocompatible nicotinic ring. Nicotine is a naturally occurring alkaloid that is best known for its use as an insecticide and a recreational drug. This exogenous molecule acts as an agonist of many nicotinic acetylcholine receptors found in the central and peripheral nervous systems.^19^ Moreover, pancreatic β-cells also present functional nicotinic receptors, which impact insulin secretion.^20^

**Scheme 1:**
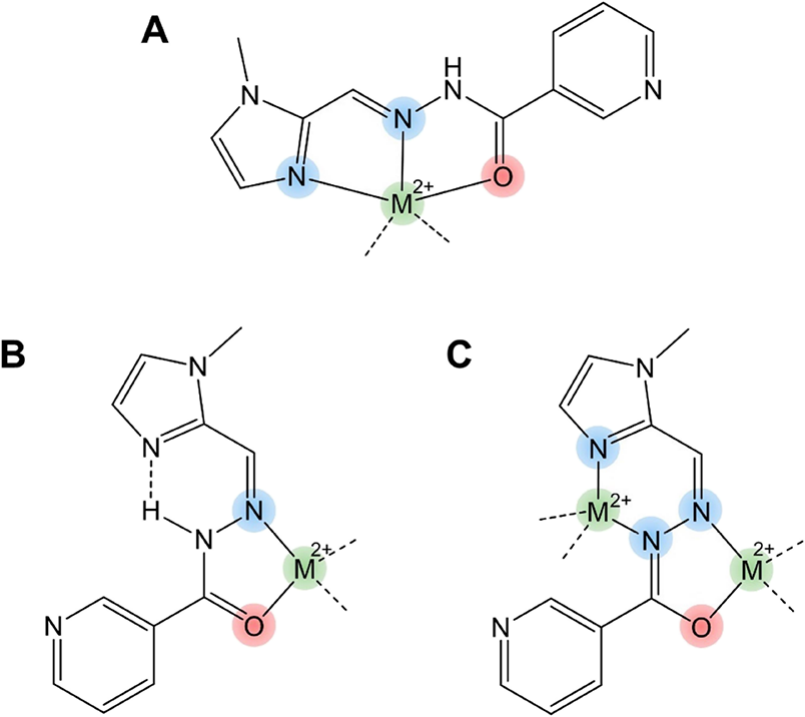
Possible Coordination Patterns for (A) Tridentate **X1NIC-(** ***E*** **)**, (B) Bidentate **X1NIC-(** ***Z*** **)**, and (C) Binucleating **X1NIC-(** ***Z*** **)** ^a^

a Metal ion and donor atoms are highlighted in color.

Although the differential biological effects of pairs of stereoisomers, including both enantiomers (e.g., thalidomide) and geometric isomers (e.g., cisplatin vs transplatin), are well-known and studied, there are no examples in the literature regarding the comparison between the properties and effects of both geometric isomers of a potentially bioactive hydrazone. Working in this direction, we separately synthesized the (*E*)- and (*Z*)-isomers of **X1NIC** and evaluated their interactions with copper­(II) ions (binary systems) as well as with the copper­(II)–hIAPP_18–22_ complex (ternary systems). The presence of histidine at position 18 makes this amylin fragment interesting from a coordination point of view.

## 2 Results and Discussion

### 2.1 Syntheses and Characterization

Two geometric, i.e., (*E*)/(*Z*), isomers of a new *N*-acylhydrazone derived from 1-methylimidazole-2-carboxaldehyde and nicotinic acid hydrazide were synthesized, which differ from each other with respect to the spatial distribution of groups around the azomethine C=N bond. **X1NIC-(** ***E*** **)** was obtained as a white solid (60% yield), after reflux for 4 h in the presence of HCl, while **X1NIC-(Z)** was isolated as a white solid (30% yield), in the absence of acid and after reflux for 24 h. The ^1^H NMR spectra of both hydrazones in DMSO-*d*_6_ show only one set of signals, indicating the obtention of isomerically pure samples (Figure S1, Supporting Information). The characteristic proton resonance of the hydrazonic amide −CONH– group of **X1NIC-(** ***E*** **)** was attributed at 13.19 ppm (Figure S1A), in agreement with the usual chemical shift region for hydrochlorides of 1-methylimidazole-containing *N*-acylhydrazones.^16,17^ The ^1^H NMR spectrum of **X1NIC-(** ***Z*** **)**, in turn, shows a highly deshielded (14.84 ppm) amide signal (Figure S1B), indicating that this proton is involved in an intramolecular H-bond with the nitrogen of the imidazole ring (see Scheme 1B), a feature that was already described by our research group for the (*Z*)-isomer of a related *ortho*-pyridine-containing *N*-acylhydrazone.^13^ These chemical shifts are similar to those found in the literature, which report that (*E*)-isomers of hydrazones usually present δ values for the amide proton at a higher field than the respective (*Z*)-isomers.^13,21^ The complete NMR signal assignment is summarized in Table S1. Moreover, ^1^H–^1^H NOESY (Figure S2) contour plots were employed in order to determine both the (*E*)/(*Z*) configurations and the *anti*-conformation.

### 2.2 Stability of the Hydrazones

Although we have already demonstrated that 1-methylimidazole-containing *N*-acylhydrazones are highly stable in aqueous solutions,^15,16^ it is important to individually assess each synthesized hydrazone to confirm such a property. Thus, we evaluated the stability of the ligands toward hydrolysis in a 1% DMSO/Tris pH 7.4 medium, at a hydrazone concentration of 5 × 10^–5^ M. No major differences were observed between the spectra of both isomers. Only one intense band, centered at 315 nm, was observed in the UV–vis spectrum of **X1NIC-(** ***E*** **)**, which can be seen in Figure 1A along with the absorptions of the precursor aldehyde and hydrazide. Absolutely no change was observed in the absorbance of the spectra taken over 12 h, demonstrating the high stability of the evaluated hydrazone. Similarly, the (*Z*)-isomer possesses only one intense absorption band centered at 319 nm (Figure 1B) and is also extremely resistant toward hydrolysis, as shown in the spectra acquired over time.

**Figure 1:**
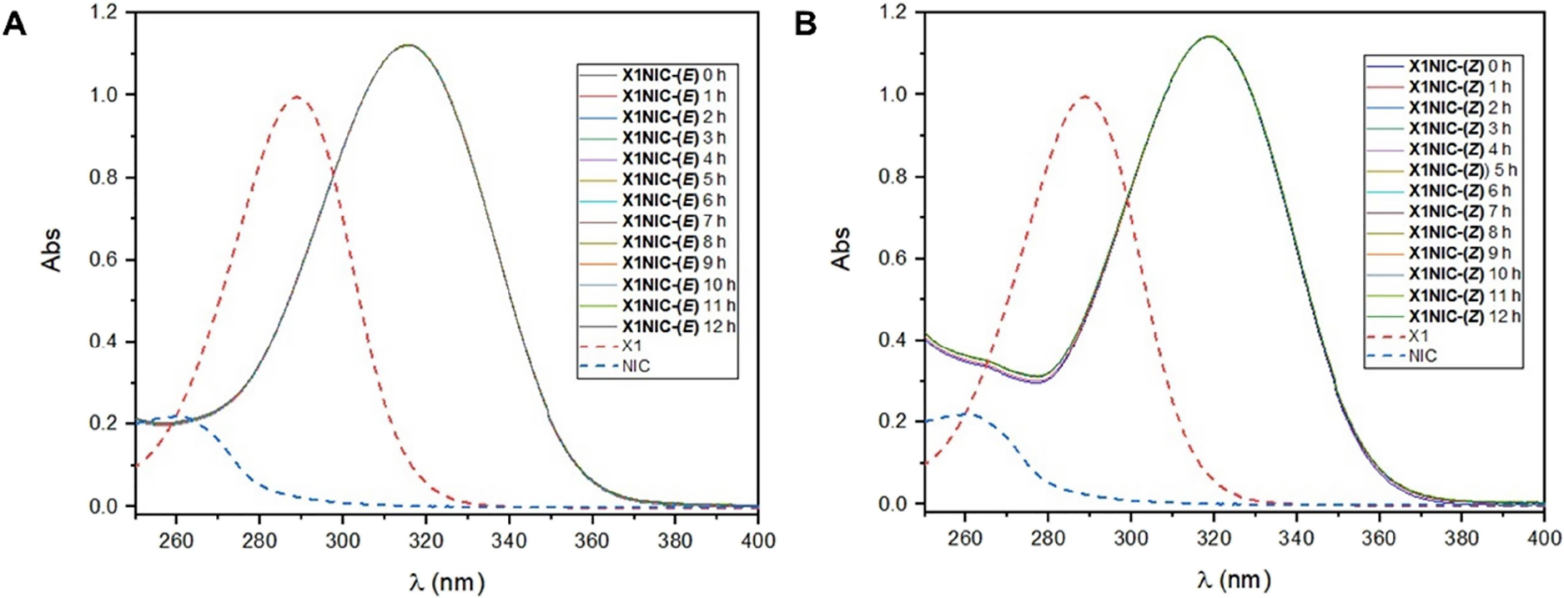
UV–vis absorption spectra of (A) **X1NIC-(** ***E*** **)** and (B) **X1NIC-(** ***Z*** **)** along with their precursors (5 × 10^–5^ M) in 1% DMSO/Tris (10 mM, pH 7.4), obtained over a period of 12 h at room temperature.

^1^H NMR spectroscopy is a powerful tool widely employed in the assessment of isomerization of compounds in solution. With this technique, we conducted a study to examine the conversion of **X1NIC** isomers as a function of the temperature. An initial measurement was performed at 25 °C [orange spectra in Figure 2, for the the (*E*)-isomer, and Figure S3, for the (*Z*)-isomer]. Then, spectra were acquired every 10 °C increase until 65 °C (yellow, purple, dark green, and blue spectra). Finally, the systems were cooled back to 25 °C, and a final spectrum (shown in red in both figures) was registered.

**Figure 2:**
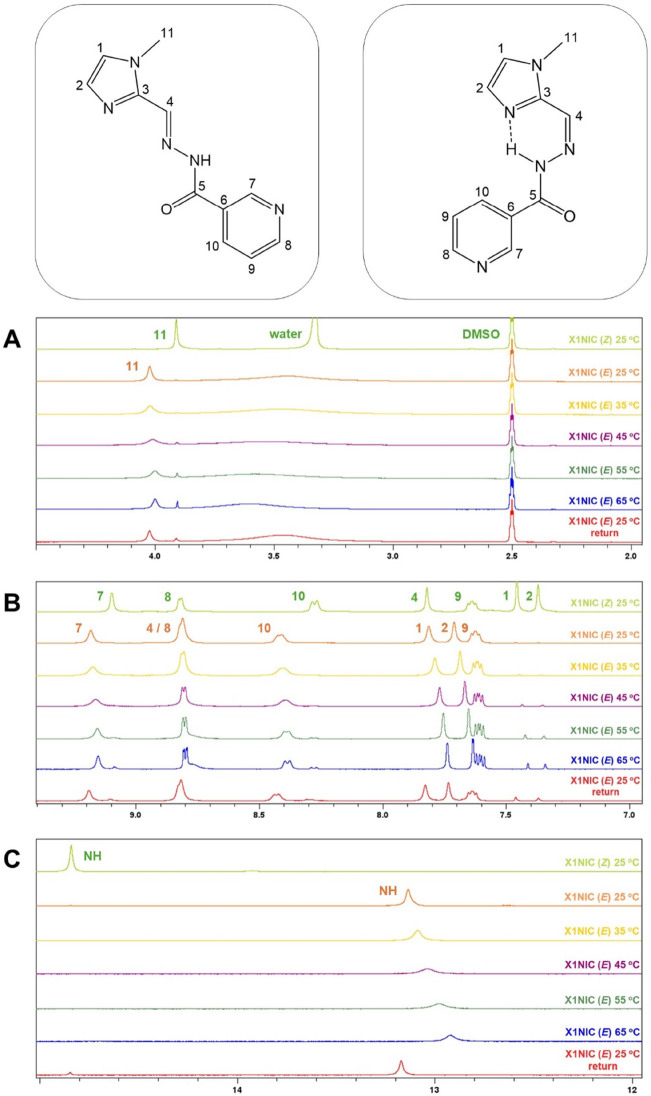
Thermal stability analysis of **X1NIC-(** ***E*** **)** assessed by ^1^H NMR between 25 and 65 °C, with spectra taken every 10 °C. A final spectrum was collected after the sample was cooled back to room temperature (25 °C). For the sake of comparison, the spectrum of **X1NIC-(** ***Z*** **)** at 25 °C was included as well. Selected spectral windows: (A) 2.0–5.0 ppm; (B) 7.0–9.5 ppm; and (C) 12.0–15.0 ppm.

We first analyzed compound **X1NIC-(*E*)**, as displayed in Figure 2. The figure also shows the spectrum of the (*Z*)-isomer at 25 °C in light green color for comparison purposes. From 45 °C on, the appearance of signals related to **X1NIC-(** ***Z*** **)** can be clearly observed. In Figure 2A, for example, which shows the spectral window of 2.0–5.0 ppm, the resonance of H11 (methyl group attached to imidazole) of the (Z)-isomer appears at 3.91 ppm. In the aromatic range of the spectra (Figure 2B), all **X1NIC-(** ***Z*** **)** signals can be detected with an increase in temperature. It is worth mentioning that the NH signal of the (*Z*)-isomer (14.84 ppm) only appears when the sample is cooled to 25 °C (Figure 2C), probably due to fast exchange with the solvent. At the highest temperature, 65 °C, the average proportions of the species in solution are 84% (*E*) and 16% (*Z*). When the temperature is brought back to 25 °C, the (*Z*)-isomer that was formed during the assay is still present, and the final solution corresponded to a mixture of both isomers.

**X1NIC-(** ***Z*** **)**, on the other hand, does not seem to undergo thermally induced isomerization (Figure S3), which indicates that this is probably the thermodynamically favored product. One possible contributing factor to this stability, although certainly not the only one, may be the presence of a strong intramolecular H-bond involving the amide hydrogen and the 1-methylimidazole group, which acts as a H-acceptor.

### 2.3 Octanol/Water Partition Coefficient

The octanol–water partition coefficient, also expressed as log *P*, is a commonly used parameter to measure a molecule’s lipophilicity in medicinal chemistry.^22^ This property is considered an important physicochemical parameter to be taken into account in the early stages of drug discovery. In this sense, the compound should be neither too hydrophilic nor too lipophilic, as this can hinder its ability to penetrate the lipid bilayers and thus be absorbed by the body.^23^ Although Lipinski’s original suggestion concerning log *P* values is that they stay below 5,^24^ guidelines regarding the specific passage of a compound through the BBB state that log *P* should be between 0 and 3.^25^

In this work, log *P* values were experimentally determined for both hydrazones using Tris buffer, pH 7.4, as the aqueous phase and 1-octanol as the organic phase. It is important to note that these compounds are expected to be neutral in the chosen (physiological) pH. The p*K*_a_ of the 1-methylimidazole nitrogen, protonated in the solid state of **X1NIC-(** ***E*** **)**, is 5.3 in this type of hydrazone,^16^ while that for the nicotinic nitrogen is around 4.4.^26^ On the other hand, the p*K*_a_ of the amide nitrogen of 1-methylimidazolic *N*-acylhydrazones is above 9.5.^16^ Thus, **X1NIC** is in its nonionic, electrically neutral form in the evaluated medium.

The values obtained for the (*E*)- and (*Z*)-isomers of **X1NIC** were 0.62 ± 0.01 and 0.87 ± 0.02, respectively. Both are in the optimal range considered for BBB crossing (0–3).^25^ Interestingly, **X1NIC-(** ***Z*** **)** is slightly more lipophilic than **X1NIC-(** ***E*** **)**, a fact that was also experimentally observed when comparing the water solubility of the compounds. The presence of an intramolecular hydrogen bond can, in fact, cause an increase in the apparent lipophilicity of the molecule.^27^ Nevertheless, both compounds still present good water solubility and a great hydrophilic–lipophilic balance.

### 2.4 Interactions with Copper­(II) Ions in Solution

The interactions of each ligand with copper­(II) ions were initially studied using the Job method and monitored through UV–vis. Representative electronic spectra of the mixtures from 0.5 to 1.0 hydrazone molar fraction (Figure 3A,B) show the intraligand absorbance [at 315 nm for **X1NIC-(** ***E*** **)** and at 319 nm for **X1NIC-(** ***Z*** **)**] that undergoes bathochromic shifts upon complexation. Interestingly, this change is much more significant for the (*E*)-isomer (372 nm) than for the (*Z*)-compound (332 nm).

**Figure 3:**
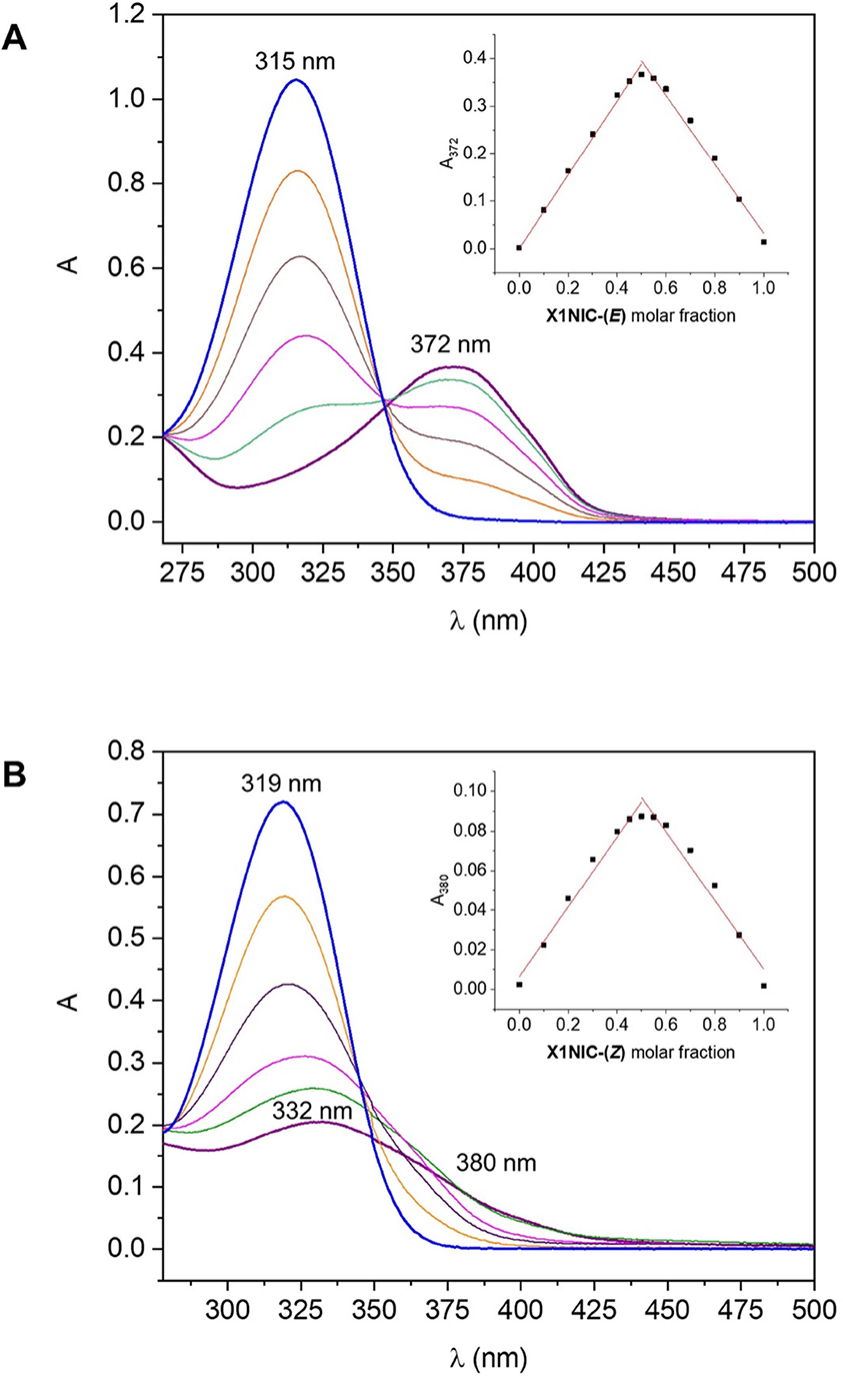
Method of continuous variations to evaluate the copper­(II) binding affinity of the two geometric isomers of **X1NIC**: (A) **X1NIC-(** ***E*** **)** and (B) **X1NIC-(** ***Z*** **)**. Representative electronic spectra of ligand molar fractions ranged from 0.5 (purple) to 1.0 (blue). Insets: absorbance versus molar fraction plots. Conditions: HEPES buffer (50 mM, pH 7.4) at 25 °C.

Regardless of this difference, a Job plot (absorbance versus molar fraction) was constructed for both hydrazones at a wavelength in which only the complex absorbs [372 nm for **X1NIC-(** ***E*** **)** and 380 nm for **X1NIC-(** ***Z*** **)**], as shown in the insets of the figure. The maximum absorbance achieved by the complexes of both isomers was at 0.5 mole fraction, which indicates that a complex with ML stoichiometry is the most stable. It is worth noting that, under these experimental conditions, no copper­(II) characteristic d–d band was observed due to the low metal concentrations employed.

Although this methodology does not provide an absolute value for the stability constants of the formed complexes, a comparison between similar compounds that were evaluated under the same experimental conditions can be performed through the calculation of an apparent constant value, *K*_app_. The compound **X1NIC-(** ***E*** **)** presented a log *K*_app_ of 5.82 ± 0.16, while **X1NIC-(** ***Z*** **)** presented a lower value: 5.04 ± 0.04, both calculated from triplicates of the experiments. The higher value obtained for the (*E*)-isomer is in perfect agreement with the fact that this compound can act as a tridentate ligand, while **X1NIC-(** ***Z*** **)** coordinates copper­(II) in a bidentate manner (Scheme 1B).

Moreover, these values can readily be compared to those of other structure-related 1-methylimidazole-containing *N*-acylhydrazones previously reported by us. For example, the mescaline-inspired X1TMP showed an apparent log *K*_app_ of 5.74 ± 0.15, while its unsubstituted analogue X1Benz presented a slightly higher log *K*_app_ value equal to 5.87 ± 0.11,^17^ both in the same range of the constant obtained for the tridentate isomer **X1NIC-(** ***E*** **)**. Another interesting comparison is that with the ligand X1INH, which has a log *K*_app_ of 5.66 ± 0.08, calculated under similar conditions.^15^ This compound is a positional isomer of the studied nicotine-inspired hydrazone and has a proven capacity to affect the aggregation state of an amyloidogenic protein in a cellular model of parkinsonism. Additionally, X1INH had its metallophoric activity demonstrated for both Cu^2+^ and Cu^+^ ions by high-field NMR spectroscopy.^15^

To further understand the affinities of these ligands toward copper­(II) ions, we performed a ^1^H NMR competition analysis on a solution containing a mixture of both isomers at a 1:1 ratio, followed by addition of a substoichiometric amount (0.05 equiv) of CuCl_2_. The presence of only 0.05 equiv of copper caused the disappearance of practically all the (*E*)-ligand signals (Figure 4). The remaining signals (most of them belonging to the (*Z*)-isomer) are marked in red in the figure, while proton attribution of the mixture prior to copper addition can be seen in orange for **X1NIC-(** ***E*** **)** and green for **X1NIC-(** ***Z*** **)**. Signals related to the 1-methylimidazole aromatic hydrogen atoms (H1 and H2) of the (*E*)-isomer, as well as the corresponding aliphatic protons (H11), are broadened beyond detection, suggesting that this ring constitutes an anchoring site for copper­(II) ions in **X1NIC-(** ***E*** **)**. The same signals, however, are not much affected in **X1NIC-(** ***Z*** **)**, supporting the bidentate nature of this isomer and suggesting that the intramolecular hydrogen bond is maintained in solution even after interaction with copper. In both cases, azomethine hydrogen (H4) and amide −NH signals are also broadened upon metal addition, due to the N,O coordination mode of the hydrazonic moiety.

**Figure 4:**
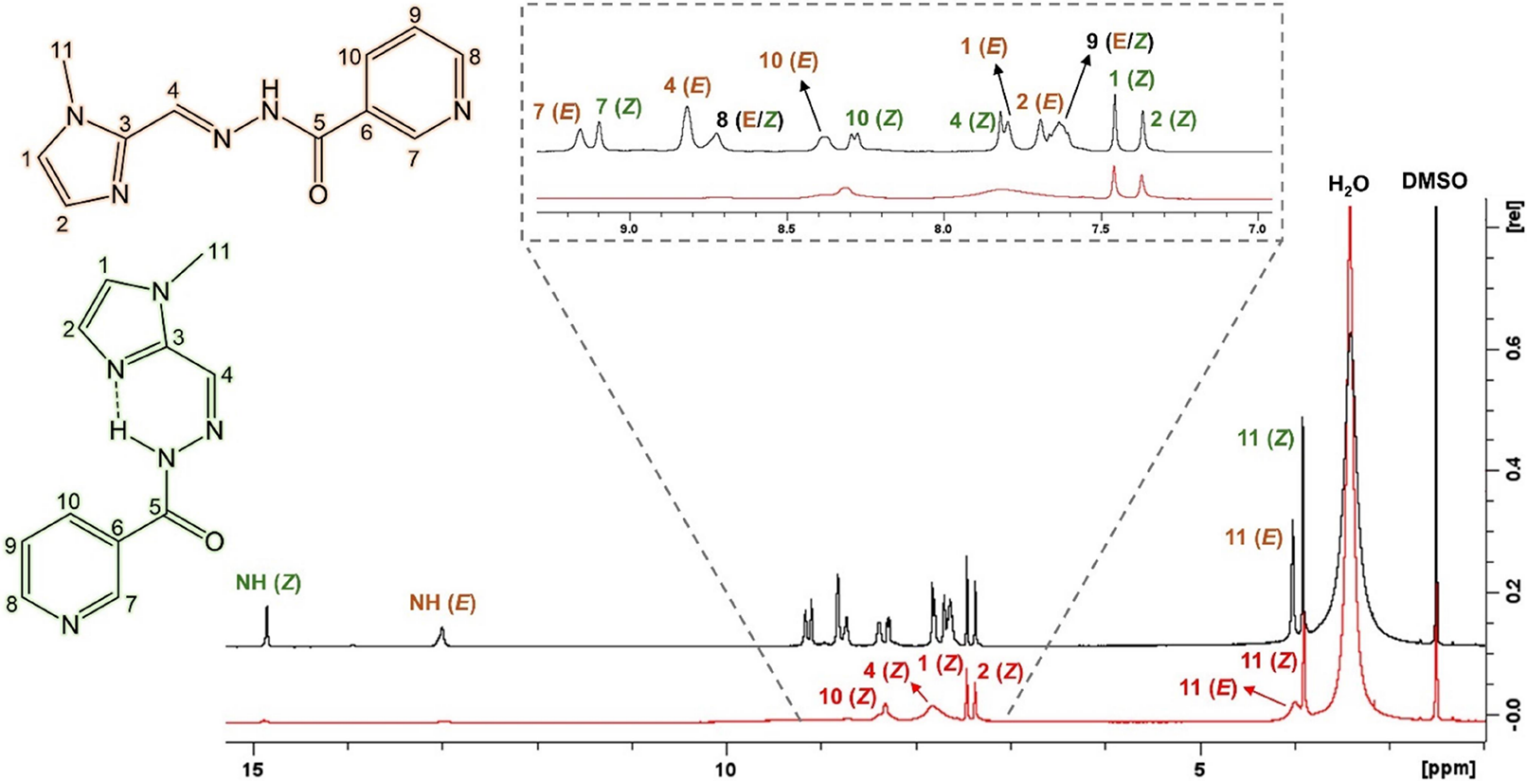
Simultaneous **X1NIC-(** ***E*** **)**/**X1NIC-(** ***Z*** **)** interactions with copper­(II) studied through ^1^H NMR (400 MHz) spectroscopy at 25 °C. Black: mixture of both **X1NIC** isomers at an *E*/*Z* ratio of 1:1, in DMSO-*d*_6_. Red: same mixture after addition of 0.05 equiv of copper­(II) in deuterium oxide, resulting in a solution containing 1% D_2_O in DMSO-*d*_6_.

### 2.5 Interactions of X1NIC Isomers with the Cu^2+^-hIAPP_18–22_ System (the Coordinating Amylin Fragment), Studied through High-Field NMR

The use of protein or peptide fragments is a valid approach to study different aspects of biologically relevant, more intricate systems and has been especially helpful in deciphering the coordinating features of amyloidogenic proteins and peptides toward endogenous metal ions such as copper­(II) and zinc­(II).^28^ This allows us, for example, to study these proteins at higher concentrations without concerns related to their aggregation. In the case of diabetes-related amylin, the hIAPP_18–22_ fragment (Scheme 2) has been proposed as the coordinating portion of the peptide^29,30^ and was employed in our studies involving the interactions of **X1NIC** isomers with the Cu^2+^-peptide system.

**Scheme 2:**
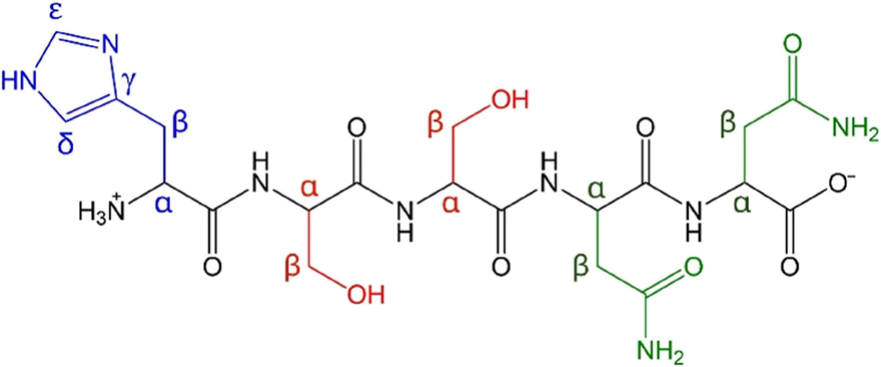
Structure of the hIAPP_18__–22_ Peptide Fragment (HSSNN) Employed in This Work^a^

a Side chains are marked in blue for H (histidine), red for S (serine), and green for N (asparagine).

First, we validated the interactions of hIAPP_18–22_ with copper­(II) ions through ^1^H NMR spectroscopy using a high-field spectrometer (700 MHz). The spectrum of the free peptide is shown in Figure 5A. The signals related to histidine (**H**18) were assigned as a triplet at 4.00 ppm (Hα), a multiplet between 3.08 and 3.02 ppm (Hβ), and two singlets at 7.01 and 7.80 ppm, associated, respectively, with the δ and ε hydrogens of the imidazole heteroaromatic ring. Regarding the two serine residues (**S**19/**S**20), their Hα signals compose a multiplet at 4.45–4.36 ppm, while the Hβ signal was attributed as another multiplet at 3.83–3.76 ppm. The −OH group was identified as a singlet at 8.51 ppm. Finally, the amide −NH_2_ protons from the side chain of the two asparagine residues (**N**21/**N**22) were observed at 7.92 and 8.39 ppm. The signals related to Hα and Hβ of these residues were attributed as multiplets at 4.45–4.36 ppm and 2.78–2.55 ppm, respectively. However, the proximity of the irradiation frequency for water suppression to those of the hydrogen nuclei affects the Hα signals of the serine and asparagine residues (**S**19/**S**20 and **N**21/**N**22), making them less intense and therefore difficult to observe.

**Figure 5:**
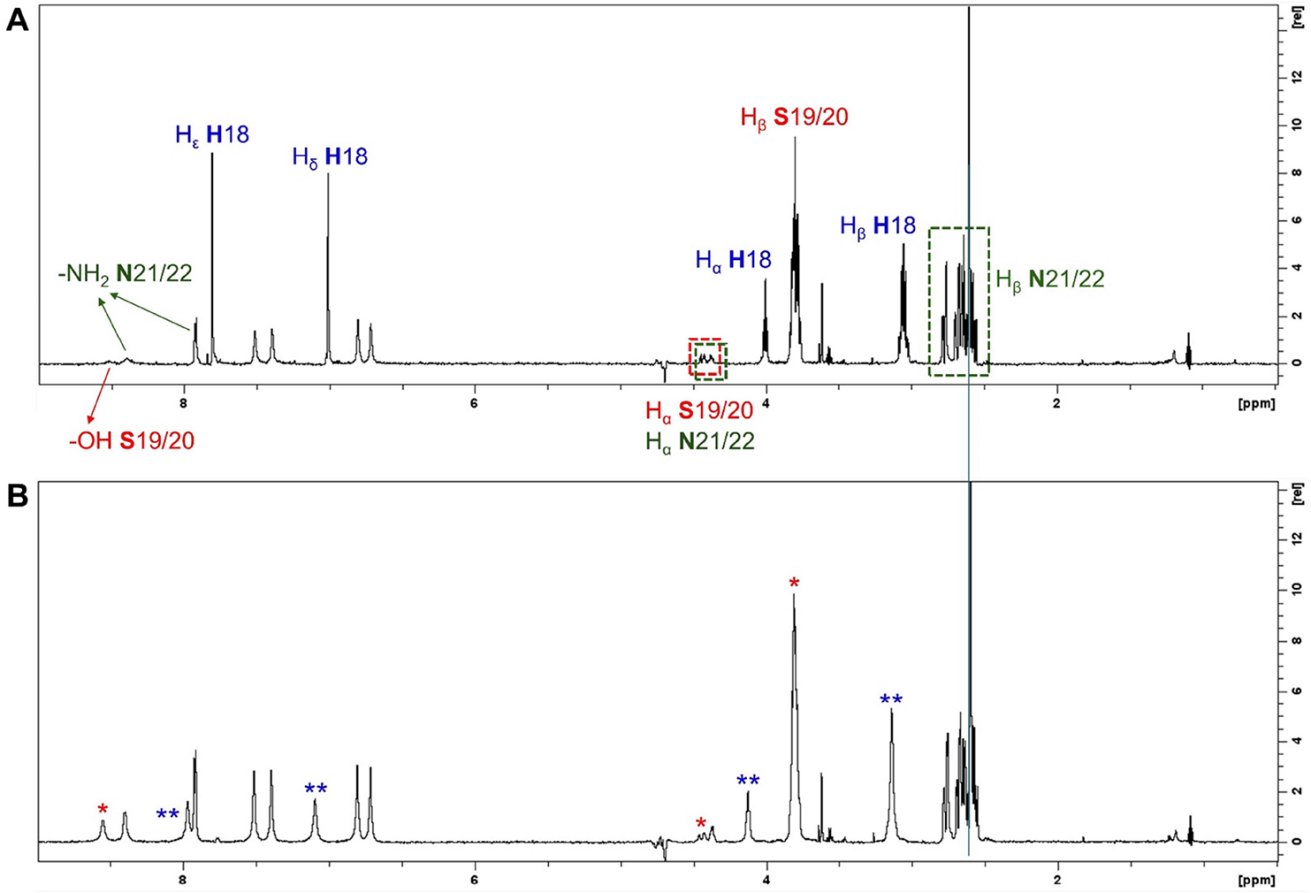
^1^H NMR spectra (700 MHz) of (A) free hIAPP_18–22_ and (B) hIAPP_18–22_ + 0.1 eq Cu^2+^, both acquired at 25 °C. Samples were prepared in 20 mM Tris-*d*_11_ at pH 7.4, with 8.5% D_2_O and 4% DMSO-*d*_6_. Histidine residues are highlighted in blue, while red was used for serine and green for asparagine. (**) indicates the most affected signals upon copper­(II) addition, while (*) highlights signals moderately modified in this context.

Figure 5B shows the ^1^H NMR spectrum of this peptide after the addition of 0.1 equiv of copper­(II). Once again, substoichiometric amounts of the metal were employed. As expected, the most affected signals (represented by an asterisk in the figure) were those of **H**18, confirming that this is the main anchoring site for the metal ion. All hydrogen atoms of this residue are deshielded, and their signals appear broadened in comparison to the spectrum of the free peptide, especially those from the aromatic ring (i.e., hydrogens δ and ε), which are directly related to the metal coordination site. Serine-related signals are also affected, although to a lesser extent (marked with * in the figure), which lead us to propose an equatorial N_3_O coordination mode similar to that reported by Rivillas-Acevedo et al.^29^ It is worth mentioning that Sánchez-López et al. demonstrated through spectroscopic analyses and theoretical studies using models of the Cu^2+^-hIAPP_18–22_ complex that coordination through the −OH side chain group is more favorable than through the carbonyl of **S**20.^30^ This is explained by the fact that the first binding is stronger, with a bond distance to copper­(II) of 2.12 Å, while the latter is weaker and distorted, with a bond distance of 2.40 Å.^30^ Finally, the signals of the asparagine residues do not appear to be affected by the addition of copper; consequently, they do not seem to be involved in coordination. Scheme 3 shows a representation of the considered Cu^2+^-hIAPP_18–22_ complex, according to the NMR data discussed above.

**Scheme 3:**
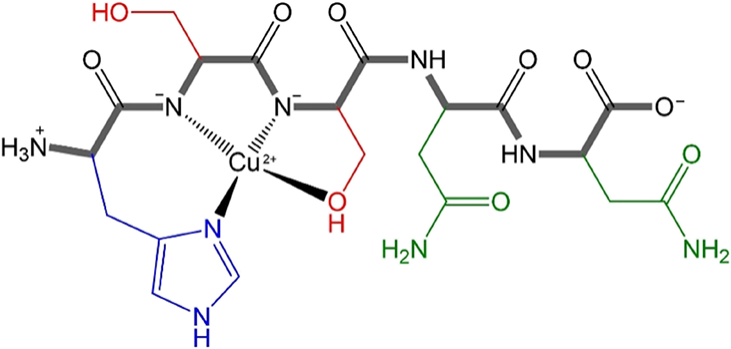
Proposed Coordination Sphere for the Neutral Cu^2+^-hIAPP_18‑22_ Complex^a^

a The peptide’s main chain is highlighted in bold gray, while the side chains are marked in blue for histidine, red for serine, and green for asparagine.

This complex was then evaluated in the presence of 0.1 and 1.0 equiv of each *N*-acylhydrazone, i.e., **X1NIC-(** ***E*** **)** and **X1NIC-(** ***Z*** **)**, as shown in Figure 6A,B, respectively. The ^1^H NMR spectra, acquired under the same experimental conditions, of each free hydrazone (black, top), the free peptide (black, bottom), and the copper­(II)-peptide complex (blue), were included for the sake of comparison.

**Figure 6:**
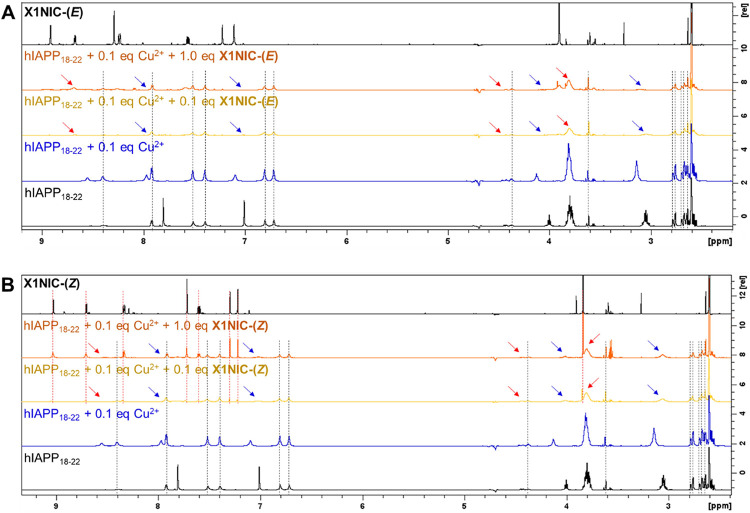
Interactions of (A) **X1NIC-(** ***E*** **)** and (B) **X1NIC-(** ***Z*** **)** with the Cu^2+^-hIAPP_18–22_ system. For each isomer, from bottom to top: ^1^H NMR spectra (700 MHz) of hIAPP_18–22_, hIAPP_18–22_ + 0.1 eq Cu^2+^, hIAPP_18–22_ + 0.1 eq Cu^2+^ + 0.1 eq compound, hIAPP_18–22_ + 0.1 eq Cu^2+^ + 1.0 equiv of compound, and free compound spectrum for comparison purposes. All spectra were acquired at 25 °C. Samples were prepared in 20 mM Tris-*d*_11_ at pH 7.4, with 8.5% D_2_O and 4% DMSO-*d*_6_. Arrows point to relevant spectral changes: red is used for changes in serine-related signals, while blue refers to histidine. Red dotted lines highlight hydrazone signals that remained unchanged. Black dotted lines, on the other hand, were used to point out unaffected peptide signals.

Interestingly, the addition of either 0.1 or 1.0 eq **X1NIC-(** ***E*** **)** not only does not recover the peptide’s signals intensities but it seems to broaden them even further, being some absorptions broadened beyond detection (dark yellow and orange spectra in Figure 6A). Blue arrows identify this effect upon histidine-related signals, while red color was used for serine. On the other hand, all signals associated with the asparagine **N**21 and **N**22 amino acid residues remained unchanged. Therefore, apart from not being involved in coordination, they also do not interact directly with the isomer **X1NIC-(** ***E*** **)**. This result is very similar to the one recently published by us for the Alzheimer’s related Aβ-Cu^2+^-X1Benz system^17^ and represents a strong indication of the formation of a ternary hIAPP_18–22_-Cu^2+^-**X1NIC-(** ***E*** **)** complex. In fact, we have already demonstrated the effect of the presence of a ternary peptide-metal-hydrazone complex on the intensity of the ^1^H NMR signals, as well as thoroughly characterized such ternary interactions with 1-methylimidazole-containing hydrazones in aqueous solution.^14,16^ It is worth noting that there is a 10-fold ligand excess with respect to copper in the 1.0 eq **X1NIC-(** ***E*** **)** sample, and the signals related to the hydrazone ligand are poorly observed, indicating fast exchange of this small molecule in the ternary complex.

Similarly to the experiment with the (*E*)-isomer, the addition of **X1NIC-(** ***Z*** **)** also caused a decrease in the intensity of histidine- and serine-related signals, and once again, metal abstraction can be ruled out in favor of the formation of a ternary hIAPP-copper-hydrazone species. However, an important difference from the previous set of experiments was observed: the appearance of **X1NIC-(** ***Z*** **)** signals, which can be seen highlighted by red dotted lines in the spectra shown in Figure 6B. At the copper:**X1NIC-(** ***Z*** **)** equimolar condition, i.e., 0.1 eq, this indicates that the interactions of the bidentate hydrazone with the Cu^2+^-hIAPP_18–22_ system are weaker than those of the (*E*)-isomer. However, when the hydrazone is present in excess (1.0 equiv) with respect to the metal ion, it shows that the exchange of the ligand with the ternary complex is quite slow, and both conditions (i.e., free and bound ligand) can be observed on the NMR time scale.

### 2.6 Cyclic Voltammetry Evaluation of Both Binary (i.e., Cu^2+^-hIAPP_18–22_ and Cu^2+^-X1NIC) and Ternary (X1NIC-Cu^2+^-hIAPP_18–22_) Systems

Since copper is an electroactive element, due to the ready availability of the +1 and +2 oxidation states in a biologically accessible potential range, we decided to follow the interactions of this metal both in the binary and ternary systems involving each **X1NIC** isomer and hIAPP_18–22_ through cyclic voltammetry (CV). As this technique is, in the present case, mainly focused on the metal, it can be considered a complementary way to confirm the conclusions taken from the ligand-oriented NMR assays.

#### 2.6.1 Cu^2+^–hIAPP_18–22_ Binary System

On one hand, the voltammogram of CuCl_2_ in buffer (50 mM HEPES pH 7.4, 100 mM NaCl) shows a quasi-reversible process at *E*_1/2_ = +160 mV vs Ag/AgCl, corresponding to the redox pair Cu^2+^/Cu^+^, as can be seen in the dotted line at the bottom of Figure 7. When in a similar medium containing 5% of the coordinating solvent DMSO, on the other hand, the quasi-reversible process of CuCl_2_ occurs at *E*_1/2_ = +90.5 mV (black line in the top of Figure 7). Another anodic process can also be seen at −53 mV. Together, this evidence suggests the existence of more than one metal-containing species under these conditions, possibly with one or more DMSO molecules coordinating copper in all of them. The use of small amounts of DMSO was necessary due to the low solubility of **X1NIC** in pure aqueous buffered medium.

**Figure 7:**
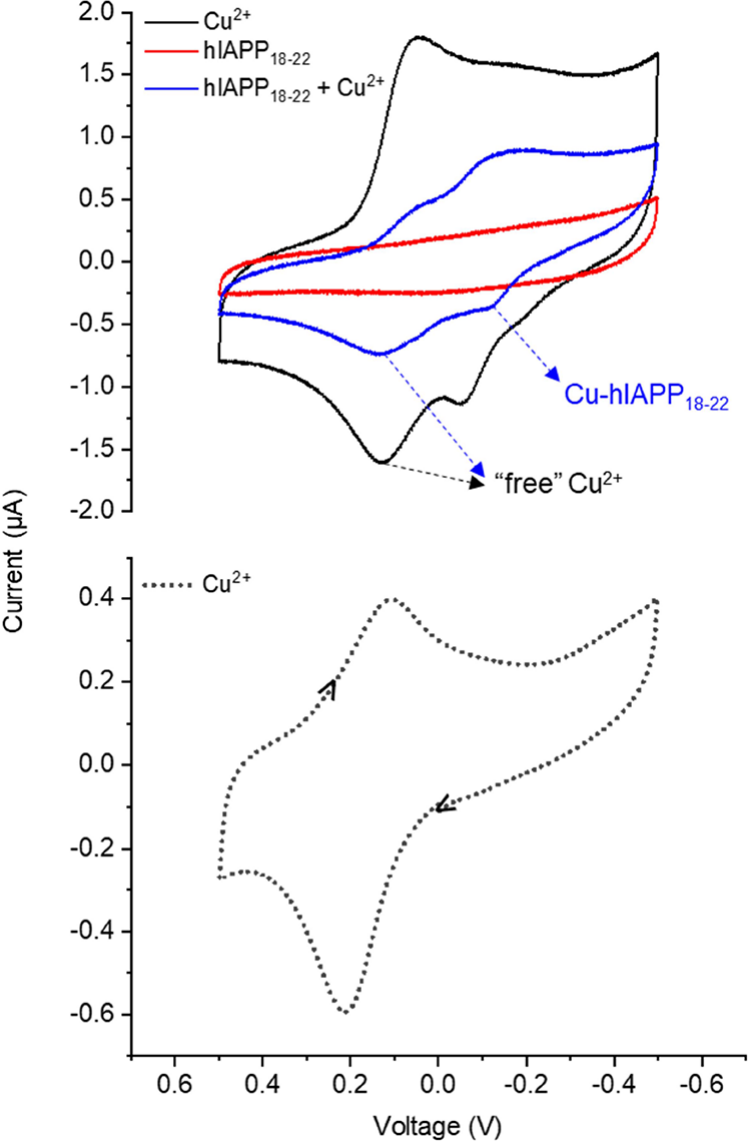
Cyclic voltammetry of the binary system involving Cu^2+^ and human IAPP coordinating fragment 18–22 (hIAPP_18–22_, HSSNN). Top: CVs of Cu^2+^ at 0.1 mM (black), hIAPP_18–22_ at 0.1 mM (red), and a 1:1 molar mixture of hIAPP_18–22_ and Cu^2+^ (blue). Conditions: 5% DMSO/buffer (50 mM HEPES pH 7.4, 100 mM NaCl) solution, at room temperature. Bottom: CV of Cu^2+^ (0.1 mM) in the absence of DMSO (50 mM HEPES at pH 7.4, containing 100 mM NaCl), at room temperature.

The peptide on its own is not electroactive, as shown by the red dotted line in Figure 7. However, in the presence of equimolar amounts of copper (blue line), the “free” metal anodic peak at −53 mV is no longer observed and, in turn, another peak at −115 mV arises. This was attributed to a process related to the binary complex Cu^2+^-hIAPP_18–22_. Nevertheless, the quasi-reversible process at *E*_1/2_ = +90.5 mV can still be seen, indicating that there are “free” metal ions remaining in solution, i.e., not bound to the peptide. This profile somewhat differs from the one reported by Seal and Dey.^31^ However, although they employed both the complete human IAPP and its N-terminal region, the latter only contained the amino acid residues from 1 to 19, which may explain such a difference, since it does not involve the same coordination set as the one in hIAPP_18–22_.

#### 2.6.2 Cu^2+^-**X1NIC** Binary Systems

Similarly to the peptide, the (*E*)-isomer of **X1NIC** is not electroactive (black line, Figure 8A). When present at a 1:1 stoichiometry with copper, two waves, one cathodic and the other anodic, can be clearly identified at potentials of −135 mV and +195 mV, respectively, as shown in yellow in the figure. These processes were attributed to the ML complex. Nevertheless, there is, once again, a small amount of “free” copper in solution, as evidenced by the residual wave indicated by arrows in the figure. Upon complexation, Cu^2+^ becomes harder to reduce in comparison to its “free” form, as can be stated by the shift of the reduction process to a more negative potential. This is in accordance with the fact that the tridentate, meridional **X1NIC-(** ***E*** **)** ligand occupies three of the four equatorial positions in the coordination sphere of the metal. Thus, in order to reduce it to the Cu^+^ state, a **X1NIC-(** ***E*** **)**–copper bond must be broken, so that the coordination sphere can be adjusted from square planar (Cu^2+^) to tetrahedral (Cu^+^). Once acting as a bidentate ligand, the rearrangement of geometry is easier for the oxidation process, which explains why this displacement is smaller for the anodic peak.

**Figure 8:**
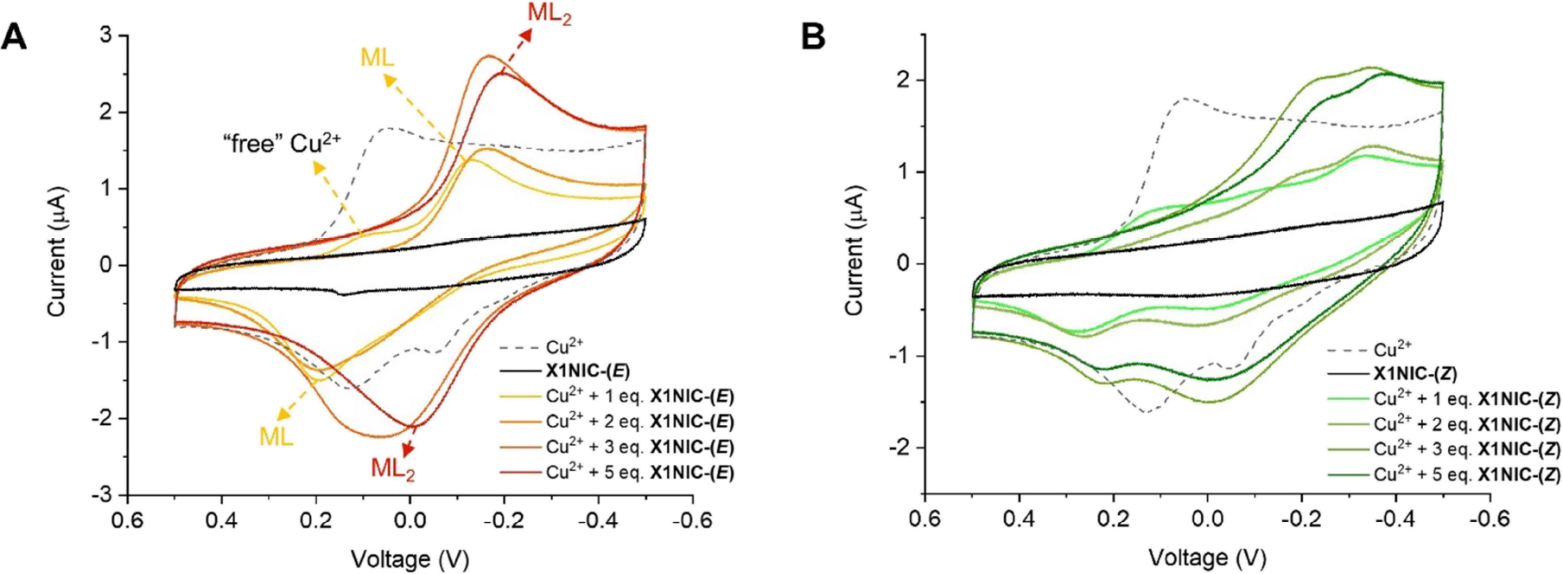
Cyclic voltammetry of binary systems involving Cu^2+^ and (A) **X1NIC-(** ***E*** **)** or (B) **X1NIC-(** ***Z*** **)**. Conditions: 5% DMSO/buffer (50 mM HEPES pH 7.4, 100 mM NaCl) solution, at room temperature. [Cu^2+^] = 0.1 mM. [hydrazone] = 0.1–0.5 mM. “Free” copper (at a concentration of 0.1 mM) voltammogram is included for the sake of comparison (gray dashed line).

Upon addition of a second equivalent of **X1NIC-(** ***E*** **)**, the remaining “free” copper appears to be completely consumed, and a mixture of ML and ML_2_ complexes composes the solution. Further increases in ligand concentration reduce the presence of the ML species, favoring an ML_2_ stoichiometry. Finally, at the 5 eq. of **X1NIC-(** ***E*** **)** end point, ML_2_ remains the only complex in solution, characterized by a quasi-reversible process at *E*_1/2_ = −102 mV (red CV in Figure 8A). It is worth mentioning that the need for a great excess of ligand to force ML_2_ formation indicates that this complex is not thermodynamically favored, which is probably related to the Jahn–Teller effect observed for copper­(II).

The same study was performed for the (*Z*)-isomer, and the voltammograms are shown in Figure 8B. Upon addition of the first equivalent of **X1NIC-(** ***Z*** **)**, “free” copper can anew be observed and seems to still be present, at a lesser amount, even after the second addition (2 equiv) of the ligand. An important feature of this system that is not seen in that of the (*E*)-isomer is the presence of two reduction (located at −242 and −350 mV) and two oxidation (at −9 and +225 mV) processes, suggesting an equilibrium involving different species generated by the substitution of labile sites in the complex. A mixture of ML and ML_2_ complexes is discarded since this pattern remains even in the presence of 5 equiv of ligand, a condition in which the ML species should no longer exist. In fact, very little variation is observed with the addition of 3 eq of **X1NIC-(** ***Z*** **)**, in which “free” copper is not present anymore, or even 5 eq. So, as indicated by other techniques discussed above, such as UV–vis and ^1^H NMR, the ligand **X1NIC-(** ***Z*** **)** only forms ML species and is very different from its tridentate (*E*)-isomer in terms of solution coordination chemistry.

#### 2.6.3 X1NIC-Cu^2+^-hIAPP_18–22_ Ternary Systems

After studying the binary systems individually by CV, we decided to evaluate the passivation ability of the hydrazones by titrating a 1:1 hIAPP_18–22_-Cu^2+^ mixture with increasing amounts of each *N*-acylhydrazone isomer. Additions corresponded to 0.1 equiv, until the 1:1:1 ternary stoichiometry was reached. A final CV was also recorded in the presence of an excess (2 equiv) of the small molecules.

Upon addition of increasing amounts of **X1NIC-(** ***E*** **)** (Figure 9A), not only the wave related to the remaining copper in solution progressively vanishes but also that of the Cu^2+^-hIAPP_18–22_ complex (represented by the anodic process at −115 mV). When the 1:1:1 stoichiometry is reached (pink voltammogram), the formation of a ternary complex can be proposed, characterized by a cathodic peak at around −175 mV and an anodic one, observed at +160 mV. Thus, we can conclude that both binary complexes, either with the (*E*)-hydrazone or with the peptide, are not individually favored, since in both cases, there is still “free” copper in solution. However, when the three components are present in equimolar amounts, this does not occur, and neither binary complex is observed in this condition as well. This is in perfect agreement with the NMR results discussed above and indicates that the binding of **X1NIC-(** ***E*** **)** and hIAPP_18–22_ to copper­(II) is a cooperative process. Finally, an excess of **X1NIC-(** ***E*** **)** (purple voltammogram) seems to be able to start to impair the interactions between Cu^2+^ and hIAPP_18–22_, as can be observed by the partial disappearance of the ternary complex-related process (at +160 mV), and the shift of the cathodic wave to more negative potentials which, by comparison to the one in the Cu^2+^-**X1NIC-(** ***E*** **)** binary system, may be possibly related to the formation of the ML_2_ species. Nevertheless, an even greater excess of hydrazone would be necessary to completely abolish metal–peptide interactions.

**Figure 9:**
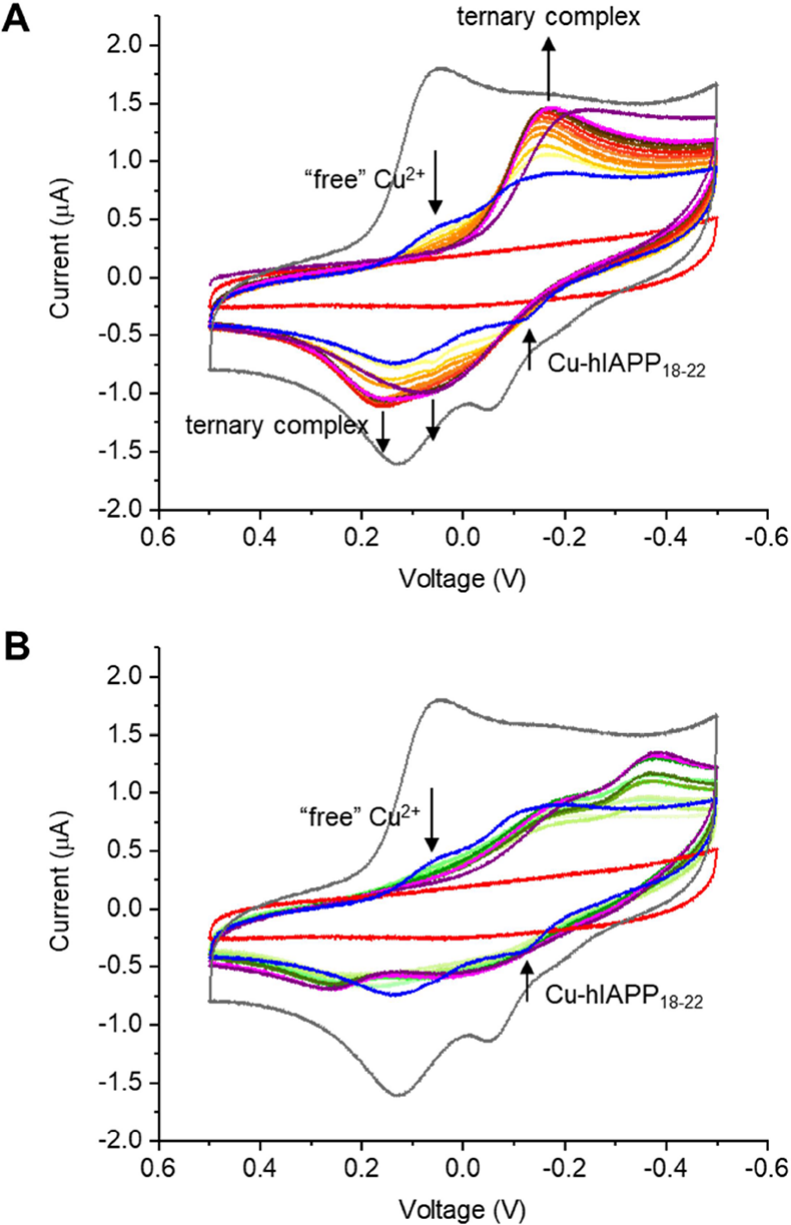
Titration of the Cu^2+^-hIAPP_18–22_ system with increasing amounts of (A) **X1NIC-(** ***E*** **)** or (B) **X1NIC-(** ***Z*** **)**. Conditions: 5% DMSO/buffer (50 mM HEPES pH 7.4, 100 mM NaCl) solution, at room temperature. Red: free hIAPP_18–22_ (0.1 mM). Gray: “free” Cu^2+^ (0.1 mM). Blue: Cu^2+^-hIAPP_18–22_ at a 1:1 stoichiometry. In (A), from yellow to dark orange: additions of 0.1 eq **X1NIC-(** ***E*** **)** (concentration range: 0.01–0.09 mM). Pink: [**X1NIC-(** ***E*** **)**] = 0.1 mM. Purple: [**X1NIC-(** ***E*** **)**] = 0.2 mM. In (B), from light to dark green: additions of 0.1 eq **X1NIC-(** ***Z*** **)** (concentration range: 0.01–0.09 mM). Pink: [**X1NIC-(** ***Z*** **)**] = 0.1 mM. Purple: [**X1NIC-(** ***Z*** **)**] = 0.2 mM.

The titration of the Cu-hIAPP_18–22_ complex with **X1NIC-(** ***Z*** **)** (Figure 9B), on the other hand, revealed a much more intricate behavior. Although the same initial results are observed, i.e., the progressive reduction of the waves related to “free” copper and the Cu^2+^-hIAPP_18–22_ complex, two, instead of one, new cathodic (−225 and −380 mV) and anodic (at +5 and +260 mV) processes arise, which are different from those observed for the binary Cu^2+^-**X1NIC-(** ***Z*** **)** system. For this reason, they were attributed to the formation of ternary species involving copper coordination by the azomethine nitrogen and carbonyl oxygen from **X1NIC-(** ***Z*** **)** and the imidazole nitrogen from the histidine residue in the hIAPP_18–22_ peptide. The fourth available equatorial position in the coordination sphere of the metal can be responsible for the presence of more than one complex in solution.

Interestingly, the addition of an excess of ligand (2 equiv, purple voltammogram) does not induce any change in comparison to the 1:1:1 system (shown in pink), indicating that the (*Z*) isomer, different from its (*E*) counterpart, cannot abstract copper­(II) from hIAPP_18–22_, and, thus, that the ternary species are somewhat stable. Once again, this is in agreement with the observations from NMR experiments, which point to an inert ternary complex when **X1NIC-(** ***Z*** **)** is involved.

To the best of our knowledge, this constitutes the first in-depth description of the interactions of the coordinating fragment hIAPP_18–22_ with copper­(II) using this technique. Moreover, this work also reveals in great detail, and for the first time via CV, the only recently proposed (by us) mechanism of action involving hydrazonic passivators, which can form stable ternary complexes at a 1:1:1 molar ratio before disrupting the anomalous metal–protein interactions when present in molar excess. Nevertheless, it is important to mention that the ternary species are already enough to partially passivate the metal, avoiding deleterious redox cycling effects.

Copper is an essential metal for the normal functioning of the body, and changes in its concentration are associated with several diseases, including diabetes.^7^ Since “free” copper can catalyze the formation of ROS through redox cycling between its two biologically available oxidation states (+1 and +2), this metal must be tightly regulated in living organisms. In the circulatory system, around 75% of copper­(II) ions are strongly bound to the copper-carrying protein ceruloplasmin, while the remaining, mostly associated with serum albumin, is considered exchangeable.^32^ Copper transport into cells, on the other hand, is mediated by CTR1. Once in the cytoplasm, copper—in its reduced state copper­(I)—is bound to the ATOX1 protein, and in healthy, physiological circumstances, any excess of the metal is excreted in bile. Under pathological conditions, however, copper can be observed bound to other proteins, including amyloidogenic peptides, such as IAPP. In this context, Qiu et al.^34^ performed a meta-analysis of plasma and serum copper concentrations and observed that patients affected by diabetes *mellitus* had higher levels of copper than healthy people, indicating that this pathology is related to an increase or improper distribution of this metal in the body. Therefore, the search for ligands that can impair such abnormal interactions, either through metal sequestering or through inactivation of its redox cycle by the formation of stable ternary species, constitutes a valid approach that certainly deserves further investigation. It has been shown that Cu^2+^-HSA can form ternary complexes with tridentate ligands such as GHK,^33^ and this supports the hypothesis that the isomers described here, particularly **X1NIC-(** ***E*** **)**, may do so as well, precluding this readily available form of copper from interacting with amyloidogenic peptides or proteins.

## 3 Conclusions

In this study, we synthesized and thoroughly characterized the geometric isomers **X1NIC-(** ***E*** **)** and **-(Z)**, new hydrazone derivatives based on a nicotinoyl scaffold, with the aim of evaluating their physicochemical properties and biological potential toward metal-associated amyloid pathology, more specifically, T2DM. We demonstrated that both isomers possess high stability in aqueous medium and lipophilicity compatible with biological applications. Both isomers were able to interact with copper­(II) ions. Complexes with different stoichiometries were obtained in solution. ML was observed in the CV and in the Job Plot experiments, with log *K*_app_ = 5.82 ± 0.16 and 5.04 ± 0.04 for **X1NIC-(** ***E*** **)** and **X1NIC-(** ***Z*** **)**, respectively. Moreover, an ML_2_ species was assigned in the CV of the (*E*)-isomer when a 5-fold excess of the ligand was present, indicating that this complex is not thermodynamically favored, which is probably related to the Jahn–Teller effect observed for copper­(II). In any case, **X1NIC-(** ***E*** **)** forms a more stable complex with Cu^2+^, which is attributed to its tridentate coordination through the hydrazone and *N*-methylimidazole moieties. These findings highlight the importance of stereochemistry in determining the coordination behavior and affinity of *N*-acylhydrazones with metal ions.

^1^H NMR and cyclic voltammetry experiments revealed that both isomers are also capable of forming ternary complexes with Cu^2+^ and the amylin fragment hIAPP_18–22_, a key sequence associated with the interaction between metals and the human islet amyloid polypeptide. These ternary species significantly altered the redox profile of the copper–peptide system, particularly by reducing the redox cycling capacity of the metal. This redox cycling is associated with oxidative stress and amyloidogenic pathways. Our results suggest that **X1NIC** compounds constitute promising chemical tools to modulate metal–peptide interactions relevant to the pathogenesis of T2DM.

The reactivity differences between the (*E*)- and (*Z*)-isomers of this hydrazone, reported for the first time in the present work, emphasize the importance of geometric isomerism in drug design, particularly in the development of passivators targeting aberrant metal–protein interactions. The ability of **X1NIC** isomers to bind biologically relevant metal–hIAPP complexes and modulate their redox activity supports the potential of *N*-acylhydrazones as leads for therapeutic intervention in T2DM. Future work will explore the effects of these ligands on full-length hIAPP aggregation and oxidative damage in cellular models to further validate their pharmacological utility. Additionally, the study of ternary interactions involving these hydrazones, copper­(II) ions, and HSA is already underway and will be the subject of an upcoming report.

## 4 Materials and Methods

### 4.1 Syntheses and Characterization

All reagents and solvents used in this work were purchased from commercial sources with the highest purity available and were employed without further purification.

The ligands were prepared through Schiff base condensation reactions between 1-methyl-2-imidazolecarboxaldehyde and nicotinic acid hydrazide at a 1:1 molar ratio, using the least volume of ethanol necessary for dissolution of each reactant. **X1NIC-(** ***E*** **)** was prepared with 1.0 mmol of reactants and 3 drops of conc. HCl, under reflux for 4 h. **X1NIC-(** ***Z*** **)**, on the other hand, was synthesized from 1.0 mmol of the reactants in the absence of acid, and the reaction mixture was kept under constant stirring and refluxed for 24 h. The precipitates were isolated through filtration after cooling of the mother liquor, washed with cold solvent, and dried at room temperature.

**HX1NIC-(** ***E*** **)** ^+^Cl^–^·1/2H_2_O (C_11_H_13_N_5_O_1.5_Cl, 274.71 g mol^–1^, M. P. = 164 ± 5 °C). IR (KBr, cm^–1^) main bands (Figure S4A): 3185 (νN^+^–H_imidazole_), 3150 (νN–H_hydrazone_), 1696 (νC=O_amide_), 1621 (νC=N_azomethine_), 1100 (νN–N).

**X1NIC-(** ***Z*** **)** (C_11_H_11_N_5_O, 229.24 g mol^–1^, M. P. = 202 ± 2 °C). IR (KBr, cm^–1^) main bands (Figure S4B): 3128 (νN–H_hydrazone_), 1685 (νC=O_amide_), 1640 (νC=N_azomethine_), 1093 (νN–N).

Melting point determinations were performed in a Fisatom model 431 apparatus in triplicate. Infrared vibrational spectroscopy (IR) was performed in a 100 FT-IR PerkinElmer spectrophotometer, and data were collected in the region 4000–400 cm^–1^. Pellets of the samples were prepared in potassium bromide. 1D ^1^H spectra and 2D ^1^H–^1^H NOESY contour maps were obtained on a Bruker Avance III HD-400 spectrophotometer (400 MHz) at 25 °C. *N*-acylhydrazones were dissolved in 0.5 mL of deuterated dimethyl sulfoxide (DMSO-*d*_6_), and the spectra were calibrated based on the residual solvent signal (quintet at 2.50 ppm).

### 4.2 Stability against Hydrolysis in Buffered Aqueous Medium

The compounds were initially prepared in 100% DMSO (stock solutions of 5 × 10^–3^ M) and then diluted to 5 × 10^–5^ M in solutions containing 1% DMSO/Tris 10 mM (pH 7.4). The spectra of the *N*-acylhydrazones were acquired at regular intervals for 12 h. A single spectrum of each precursor was registered under the same solvent and concentration conditions for comparison purposes. All spectra were acquired between the wavelengths of 200 and 800 nm at room temperature in an Agilent Cary 100 spectrophotometer.

### 4.3 Thermal Stability of the Isomers

Both isomers were prepared at 0.018 M [0.0025 g for **X1NIC-(** ***E*** **)** and 0.0020 g for **X1NIC-(** ***Z*** **)**] in 500 μL of DMSO-*d*_6_ and transferred separately to 5 mm NMR tubes. ^1^H NMR spectra (Bruker Avance III HD-400) were acquired, for each isomer, at different increasing temperatures (25, 35, 45, 55, and 65 °C). A final spectrum was then obtained after cooling the sample from 65 back to 25 °C.

### 4.4 Experimental Octanol–Water Partition Coefficient

The distribution coefficient (*P*) in the 1-octanol/water system was calculated by using the shake flask method. Tris buffer pH 7.4 (10^–2^ M) was used as the aqueous phase. The organic and aqueous phases were prepared by containing low concentrations of hydrazone (5 × 10^–5^ M). Analyses were performed in triplicate, and *P* was calculated as the average concentration ratio *C*_o_/*C*_w_, in which *C*_o_ is the final concentration in the organic phase, and *C*_w_ is the final concentration in the aqueous phase. Concentrations were estimated from calibration curves measured in an Agilent Cary 100 spectrophotometer.

### 4.5 Method of Continuous Variations

The apparent affinity of both ligands for copper­(II) ions and the stoichiometry of the reactions were evaluated, in solution, using the Method of Continuous Variations (Job’s method) monitored through UV–vis (Agilent Cary 100 spectrophotometer), in triplicate. While this method is mainly used for determining complex stoichiometries, under some specific conditions, such as the formation of only one complex species, little or no overlapping of free and complexed ligand bands, and strong metal–ligand affinity, an apparent constant (*K*_app_) can be obtained from the data.^15^ To do so, initially, the molar absorptivity of the ligand was determined through a calibration curve in HEPES buffer (50 mM, pH 7.4). Then, mixtures of different ligand to metal molar fractions were prepared as usual from stock solutions of each ligand and CuCl_2_·2H_2_O, also in HEPES, at a concentration of 5 × 10^–5^ M. The mixtures were stirred at 25 °C and 500 rpm until the moment of analysis. A “theoretical” molar absorptivity for the formed complex was calculated at the point of intersection of the lines using the Lambert–Beer equation. The complex concentration at the equilibrium was then estimated from this ε value according to the maximum experimental absorbance of its band. The free ligand concentration at equilibrium, on the other hand, was determined from the remaining absorbance of the hydrazone band in the sample of molar fraction related to the observed stoichiometry. Finally, an apparent affinity constant can be characterized considering the equilibrium concentrations of the complex, ligand, and metal (calculated taking into account the reaction stoichiometry) by the fundamental equation $K_{\mathrm{app}}=\frac{\left[\mathrm{ML}\right]}{\left[L][M\right]}$ , valid for a 1:1 complex.

### 4.6 Simultaneous Cu^2+^ Interactions with X1NIC-(*E*) and X1NIC-(*Z*)

Stock solutions of the ligands were prepared at 0.025 M [0.0021 g for **X1NIC-(** ***E*** **)** and 0.0017 g for **X1NIC-(** ***Z*** **)**] in 300 μL DMSO-*d*_6_ each. 0.0021 g of CuCl_2_·2 H_2_O was dissolved in 200 μL D_2_O, attaining a stock concentration of 0.0617 M. In the same 5 mm NMR tube, 250 μL of each isomer was added, and a ^1^H spectrum (Bruker Avance III HD-400) of this mixture was acquired for comparison purposes. Then, 5 μL (0.05 equiv) of the metal solution was added, and another ^1^H spectrum was recorded. Measurements were performed at 25 °C.

### 4.7 ^1^H NMR of the Cu^2+^-hIAPP_18–22_ and the Cu^2+^-hIAPP_18–22_-X1NIC System

All NMR spectra of the hIAPP_18–22_ fragment peptide (GenScript, amino acid sequence HSSNN·2 TFA, molecular weight 785.56 g mol^–1^) were collected at 25 °C on a Bruker Avance III 700 MHz spectrometer using a TXI inverse detection triple resonance probe (^15^N, ^13^C, and ^1^H). This equipment is located in the Jiri Jonas National Nuclear Magnetic Resonance Center from the National Center for Structural Biology and Bioimaging (CENABIO), UFRJ, Rio de Janeiro, Brazil. A 20 mM Tris-*d*_11_ buffer was prepared in ultrapure water, adjusted to pH 7.4, and filtered with the help of a 0.22 μm syringe filter. Then, 0.5 mg of the peptide was dissolved in 520 μL of this buffer, with the addition 50 μL of D_2_O and 25 μL of DMSO-*d*_6_, resulting in a 1.0 mM peptide solution, which was transferred to a 5 mm NMR tube. A ^1^H spectrum was initially acquired for the free hIAPP_18–22_, then with the addition of 0.1 equiv of Cu^2+^, from a stock solution of 0.0318 mM CuCl_2_ · 2 H_2_O in D_2_O. After that, samples were measured in the presence of 0.1 and 1.0 equiv of either **X1NIC-(** ***E*** **)** or **X1NIC-(** ***Z*** **)**. Ligands were prepared as 0.0636 M DMSO-*d*_6_ stock solutions. All spectra were acquired and processed by using TopSpin 3.5 software (Bruker).

### 4.8 Cyclic Voltammetry (CV) of the Cu^2+^-hIAPP_18–22_ and the Cu^2+^-hIAPP_18–22_-X1NIC Systems

Electrochemical analyses were performed on a Basi Epsilon potentiostat with a C3 cell holder. A three-electrode system was used that was composed of a glassy carbon working electrode, a Ag/AgCl reference electrode, and a platinum auxiliary electrode. The hIAPP_18–22_ peptide (GenScript, amino acid sequence HSSNN·2 TFA, molecular weight 785.56 g mol^–1^) stock solution (0.5 mM) was prepared in 50 mM HEPES pH 7.4, with 100 mM NaCl. The CuCl_2_·2H_2_O stock solution was prepared at a concentration of 5 mM in ultrapure water. Stock solutions of the ligands were also made at a concentration of 5 mM, but in anhydrous DMSO, due to the low solubility of the (*Z*)-isomer. The analyses were performed with 3 mL of buffer containing 5% anhydrous DMSO in the cell. The peptide was diluted to a final concentration of 0.1 mM. Then, 1 equiv of Cu^2+^ was added, followed by the titration of the ligands from 0.1 to 2 equiv. CVs were collected at a scan rate of 100 mV s^–1^ over a potential range of 500 to −500 mV, under a nitrogen atmosphere.

## Supplementary Material



## Notes

^1^H NMR spectra and assignment for **X1NIC-(** ***E*** **)** and **X1NIC-(** ***Z*** **)**, along with their ^1^H–^1^H NOESY NMR spectra; thermal stability analysis of **X1NIC-(** ***Z*** **)** as well as the mid-infrared spectra of both isomers (PDF)

## ∥

In memoriam.
