# Supplementary material for: Differential Modulation of Copper(II) Interactions with the 18–22 Coordinating Amylin Fragment by the Geometric Isomers of a New Nicotinoyl Hydrazone: A First Study
Source: ACS Omega. 2025 Jul 11;10(28):31115–27. doi: 10.1021/acsomega.5c04850 (PMC12290932; doi:10.1021/acsomega.5c04850)
Supplement: Supplementary file 1 [file ao5c04850_si_001.pdf]

Differential modulation of copper(II) interactions with the 18-22 coordinating amylin fragment by the geometric isomers of a new nicotinoyl hydrazone: a first study

Alessandra Carvalho[1], Karina C. Pougy[2], Anderson S. Pinheiro[2†], Daphne S. Cukierman[3*], Nicolás A. Rey[1*]

[1] Department of Chemistry, Pontifical Catholic University of Rio de Janeiro (PUC-Rio), Rio de Janeiro, 22451-900, Brazil

[2] Department of Biochemistry, Institute of Chemistry, Federal University of Rio de Janeiro (UFRJ), Rio de Janeiro, 21941-909, Brazil

[3] Department of General and Inorganic Chemistry, Institute of Chemistry, State University of Rio de Janeiro (UERJ), Rio de Janeiro, 20550-013, Brazil

[†]*in memoriam*

*Joint corresponding authors:
Prof. Daphne S. Cukierman – dcukierman.uerj@gmail.com
Prof. Nicolás A. Rey – nicoarey@puc-rio.br – (+55 21) 3527-1813

Supporting Information

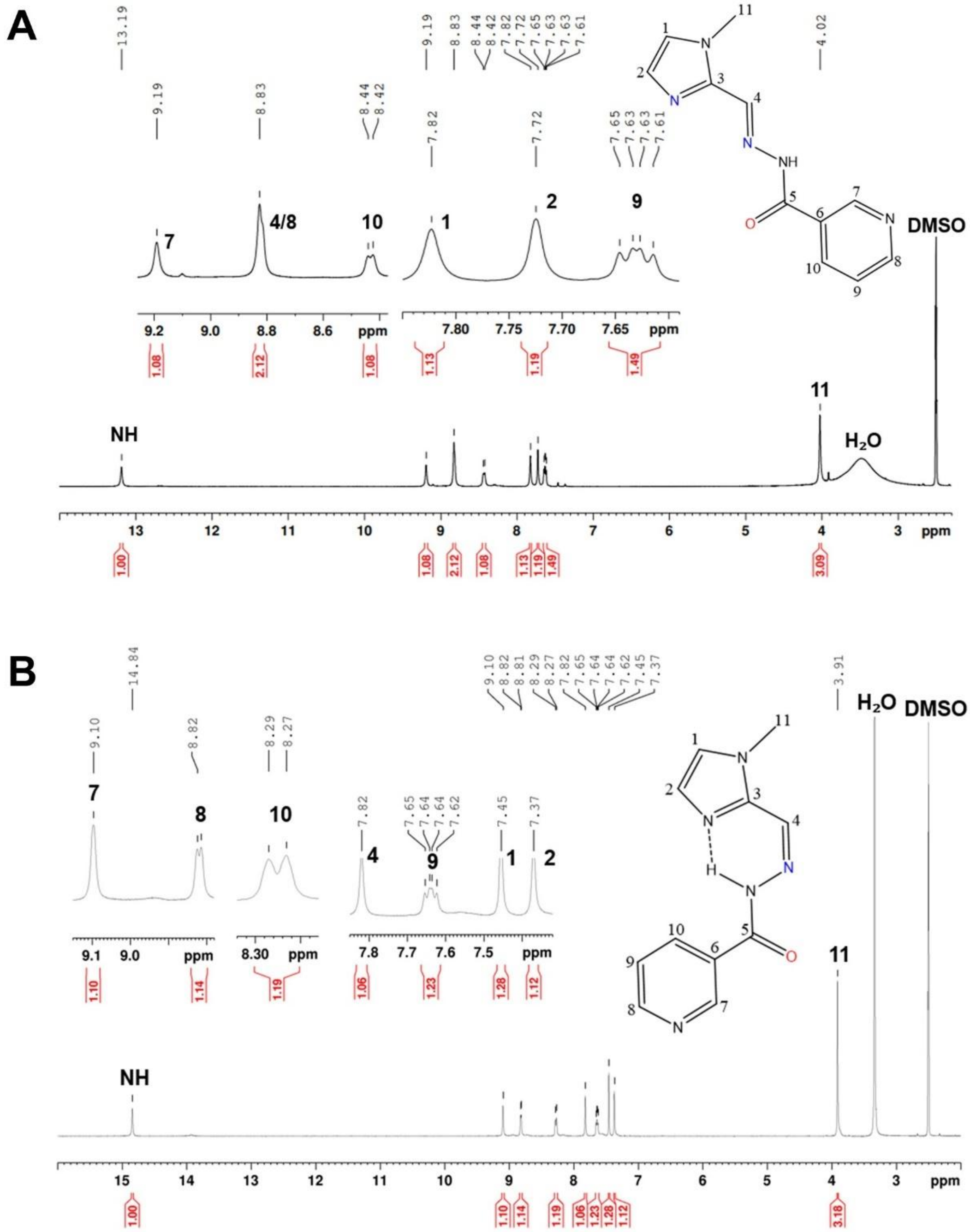

Figure S1. ^{1}H NMR spectrum (400 MHz) of (**A**) **X1NIC-(*E*)** and (**B**) **X1NIC-(*Z*)** in DMSO-d_6 at 25 °C.

Table S1. ^{1}H (400 MHz) signal attribution for **X1NIC-(*E*)** and **X1NIC-(*Z*)** (DMSO-d_6 at 25 °C).

	**X1NIC-(*E*)**	**X1NIC-(*Z*)**
**H**	**δ (ppm)**	**δ (ppm)**
**1**	7.82 (s, 1H)	7.45 (s, 1H)
**2**	7.72 (s, 1H)	7.37 (s, 1H)
**4**	8.83 (s, 1H)	7.82 (s, 1H)
**7**	9.19 (s, 1H)	9.10 (s, 1H)
**x8**	8.83 (d, 1H)*	8.82 (d, 1H, $^3J_{HH}$ = 4.50 Hz)
**9**	7.63 (dd, 1H $^3J_{HH}$ = 4.96 Hz, $^3J_{HH}$ = 7.16 Hz)	7.64 (dd, 1H, $^3J_{HH}$ = 4.50 Hz, $^3J_{HH}$ = 7.48 Hz)
**10**	8.43 (d, 1H, $^3J_{HH}$ = 7.16 Hz)	8.28 (d, 1H, $^3J_{HH}$ = 7.48 Hz)
**11**	4.02 (s, 3H)	3.91 (s, 3H)
**NH**	13.19 (s, 1H)	14.84 (s, 1H)

Signal multiplicity: (s: singlet; d: doublet; dd: doublet of doublets).
(*) doublet is overlap with H4.

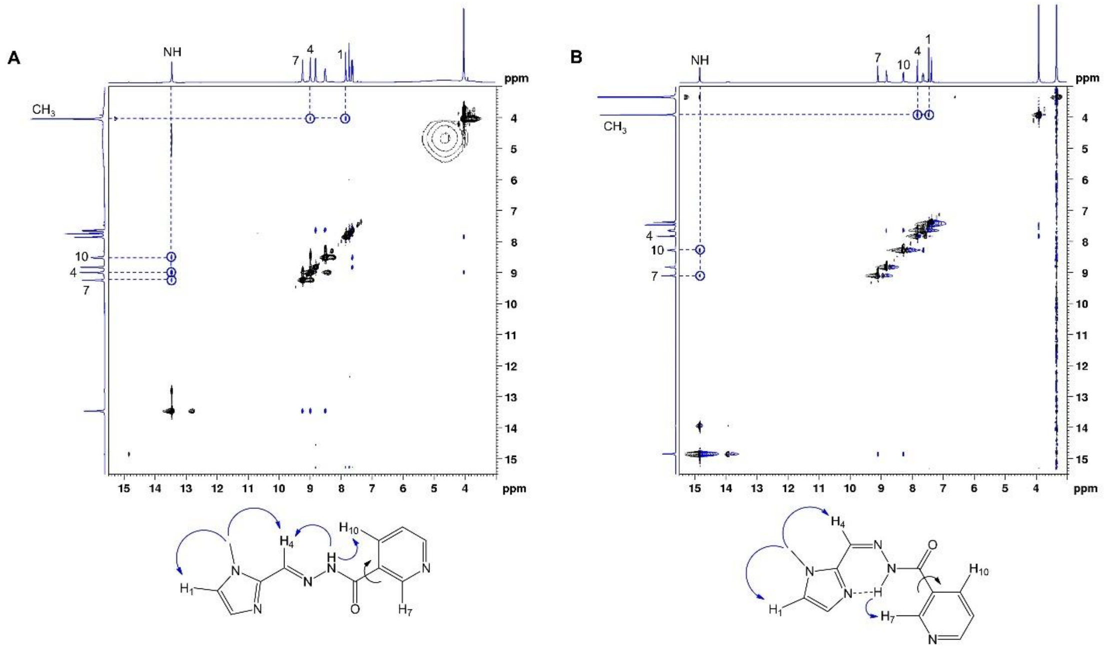

Figure S2. ^{1}H-^{1}H NOESY NMR spectrum (400 MHz) of (**A**) **X1NIC-(*E*)** and (**B**) **X1NIC-(*Z*)** in DMSO-d_6 at 25 °C (top). The proposed structure of the isomers in anti-conformations and their spatial correlations are shown on the bottom. Blue arrows indicate relevant expected dipolar couplings.

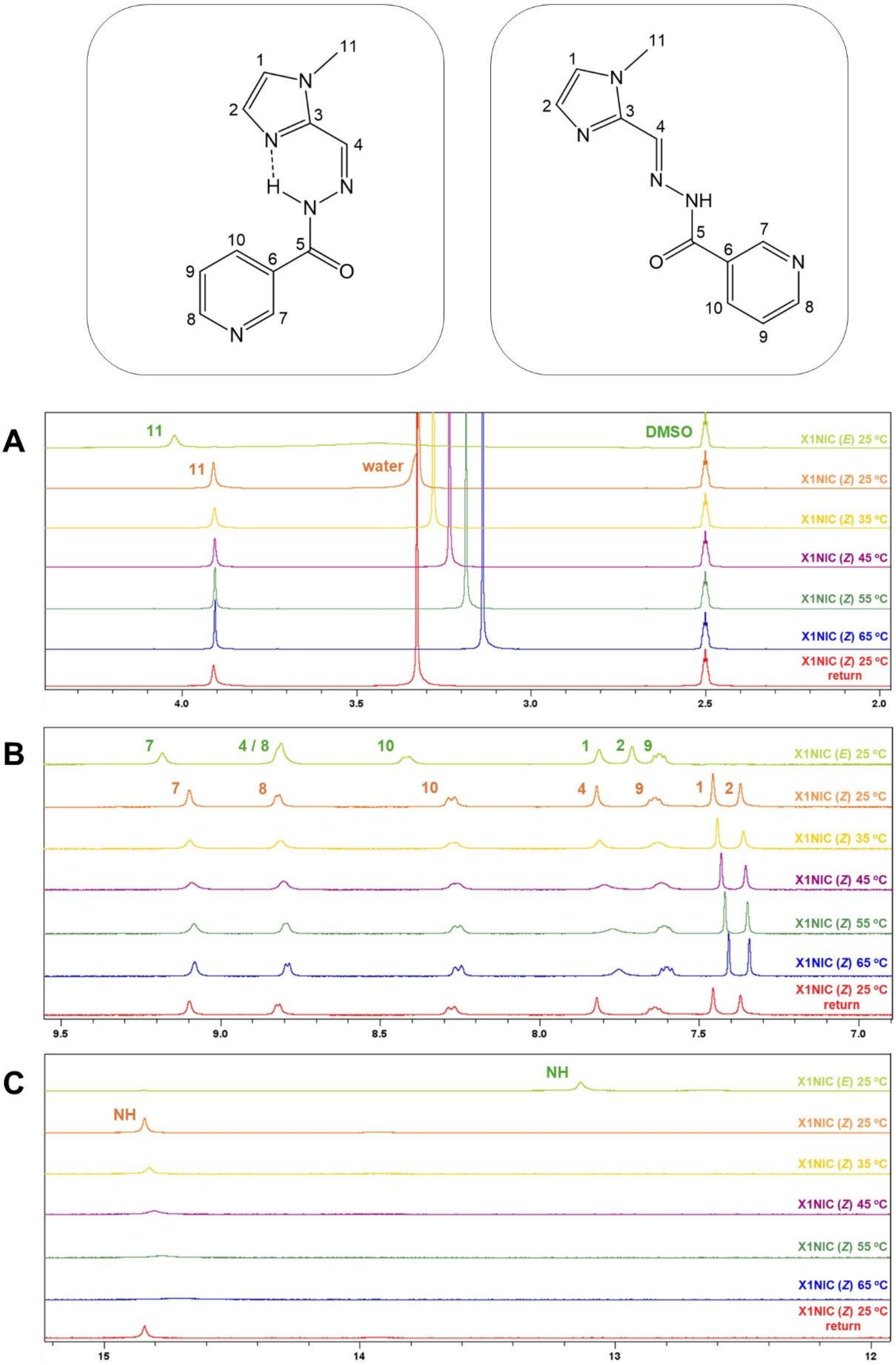

Figure S3. Thermal stability analysis of **X1NIC-(*Z*)** assessed by ^{1}H NMR, with spectra taken every 10 ºC, from 25 to 65 ºC. A final spectrum was collected after cooling the sample back to room temperature (25 ºC). Selected spectral windows: (**A**) 2.0 – 5.0 ppm; (**B**) 7.0 – 9.5 ppm; (**C**) 12.0 – 15.0 ppm.

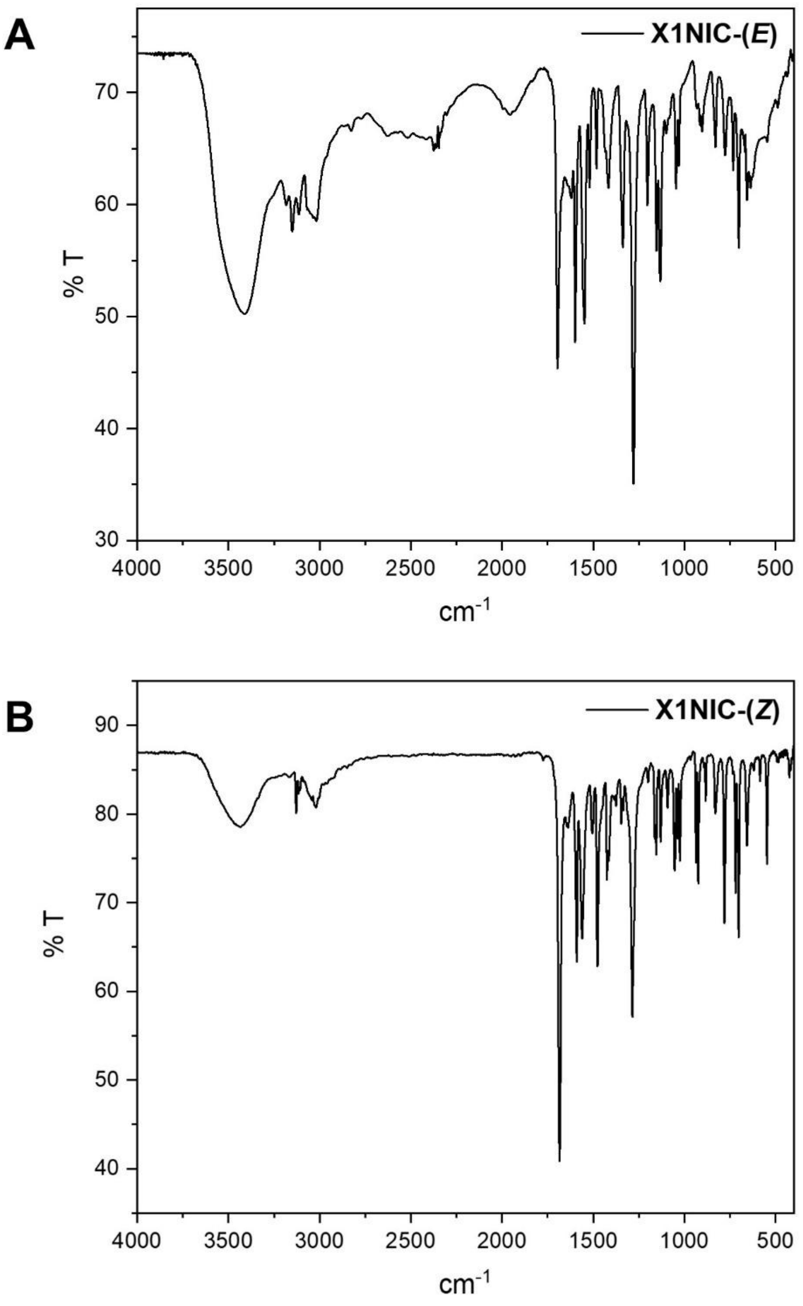

Figure S4. Mid-infrared spectra of (**A**) **X1NIC-(*E*)** and (**B**) **X1NIC-(*Z*)**. Samples were prepared as KBr pellets.
